# Transcriptome Profiling of Rabbit Parthenogenetic Blastocysts Developed under *In Vivo* Conditions

**DOI:** 10.1371/journal.pone.0051271

**Published:** 2012-12-12

**Authors:** Carmen Naturil-Alfonso, María dels Desamparats Saenz-de-Juano, David S. Peñaranda, José S. Vicente, Francisco Marco-Jiménez

**Affiliations:** Instituto de Ciencia y Tecnología Animal, Universidad Politécnica de Valencia, Valencia, Spain; Justus-Liebig-Universität, Germany

## Abstract

Parthenogenetic embryos are one attractive alternative as a source of embryonic stem cells, although many aspects related to the biology of parthenogenetic embryos and parthenogenetically derived cell lines still need to be elucidated. The present work was conducted to investigate the gene expression profile of rabbit parthenote embryos cultured under *in vivo* conditions using microarray analysis. Transcriptomic profiles indicate 2541 differentially expressed genes between parthenotes and normal *in vivo* fertilised blastocysts, of which 76 genes were upregulated and 16 genes downregulated in *in vivo* cultured parthenote blastocyst, using 3 fold-changes as a cut-off. While differentially upregulated expressed genes are related to transport and protein metabolic process, downregulated expressed genes are related to DNA and RNA binding. Using microarray data, 6 imprinted genes were identified as conserved among rabbits, humans and mice: *GRB10*, *ATP10A*, *ZNF215*, *NDN*, *IMPACT* and S*FMBT2*. We also found that 26 putative genes have at least one member of that gene family imprinted in other species. These data strengthen the view that a large fraction of genes is differentially expressed between parthenogenetic and normal embryos cultured under the same conditions and offer a new approach to the identification of imprinted genes in rabbit.

## Introduction

Embryonic stem cells (ESCs) have enormous potential in biomedicine for cell replacement, drug screening, predictive toxicology and developmental studies [1] and are envisaged as a powerful source of pluripotent cells for differentiation into desirable tissue for regenerative medicine and cell therapy [2], [3]. Despite the tremendous potential of ESCs, their handicap is the isolation method, as they are obtained from the inner cell mass of a blastocyst, making the embryo unviable [4].

Parthenogenetic embryos are being studied as an alternative source of ESCs, which would avoid ethical concerns related to destruction of the embryo [4], [5]. ESCs derived from parthenogenetic embryos (pESCs) have been shown to differentiate into all cell types and functional organs in the body [6]. However, several studies have evaluated similarities and differences between parthenogenetic and conventional ESCs in pluripotency, karyotype, *in vivo* and *in vitro* differentiation ability and RNA expression levels in human, nonhuman primates and rabbit [1], [2], [3], [5], [7], [8]. Generally, they present normal karyotypes and are similar in their undifferentiated state, expressing normal pluripotency markers, but present different transcriptomes, with different expression patterns of extracellular matrix proteins and methylation.

In rabbit, ESCs lines from different origin have been derived and characterised [8], [9]. Fang et al. [8] showed that ESCs derived from fertilised, parthenogenetic and nuclear transfer embryos seem to be similar, in that all three types were able to give rise to cells and tissue types of the three primary germ layers when ESCs are cultured *in vivo* and *in vitro*. In this case, ESCs of parthenogenetic and nuclear transfer embryos were derived using the same protocol. However, the origin of the source of the cell line has important consequences [1]. Piedrahita et al. [10] showed that ESCs lines from mice and pigs derived with the same protocol have some similar characteristics, but not all. Under *in vitro* culture, parthenote embryos present altered mRNA expression patterns, while *in vivo* developed parthenotes seem to be similar to normal embryos for the expression of factor *OCT-4*, Vascular Endothelial Growth Factor, Epidermal Growth Factor Receptor 3 and Transforming Growth Factor β2 genes [11]. In fact, in parthenote embryos the maximum development reached in all mammals species has been reported when embryos were transferred to subrogate females in early stages of development, providing a large *in vivo* culture.

In the present work, we employed a microarray to characterise transcriptome differences between 6-day parthenote embryos and 6-day fertilised blastocysts developed *in vivo.* In addition, based on the list of candidate genes identified by microarray, we studied the expression levels of selected transcripts in the parthenotes and fertilised blastocyst derived *in vivo* and checked this list with a database of genes previously listed as imprinted, while also reporting the identification of putative imprinted genes in rabbit blastocysts.

## Materials and Methods

All chemicals in this study were purchased from Sigma-Aldrich Química S.A. (Madrid, Spain) unless stated otherwise.

### Animals

Mature (adult) rabbit does belonging to the New Zealand White line from the ICTA (Instituto de Ciencia y Tecnología Animal) at the Polytechnic University of Valencia (Spain) were used as oocyte and embryo donors and recipient does. The Ethics and Animal Welfare Committee of the Universidad Politécnica de Valencia approved this study. All animals were handled according to the principles of animal care published by Spanish Royal Decree 1201/2005 (BOE, 2005; BOE = Official Spanish State Gazette).

### Parthenogenetic oocyte activation

To obtain oocytes for parthenogenetic activation, 32 receptive does were induced to ovulate with an intramuscular dose of 1 µg of Buserelin acetate. Does were slaughtered 16–18 h post-induction of ovulation and the reproductive tract was immediately removed. Oocytes were recovered by perfusion of each oviduct with 5 mL of pre-warmed Phosphate Buffered Saline without calcium chloride (PBS) and supplemented with 0.1% of Bovine Serum Albumin (BSA). Recovered oocytes were submitted to two sets 1 h apart of two DC electrical pulses of 3.2 kv/cm for 20 µs at 1 sec apart in an activation medium (0.3 M mannitol supplemented with 100 µM MgSO_4_ and 100 µM CaCl_2_), followed by 1 h exposure in TCM199 medium supplemented with 5 µg/µL of cycloheximide and 2 mM of 6-DMAP. A total of 369 oocytes were activated.

### Oviductal transfer by laparoscopy

Presumptive parthenotes were transferred by laparoscopy into oviducts of 13 synchronised receptive does just after activation, whose ovulation was induced as previously described [12], [13]. About 28 activated oocytes per doe were transferred. Receptive does were anaesthetised by an intramuscular injection of 16 mg xylazine (Rompun; Bayern AG, Leverkusen, Germany), followed by an intravenous injection of ketamine hydrochloride at the rate of 25 mg/kg body weight (Imalgene 1000; Merial S.A, Lyon, France) to keep does under anaesthesia during laparoscopy. Females were slaughtered 6 days later and parthenote blastocysts were recovered by uterine horns perfusion with 20 mL of Dulbecco Phosphate Buffered Saline (DPBS) supplemented with 0.1% of BSA.

### Control embryo recovery at day 6 of development

Six receptive does were artificially inseminated with pooled sperm from fertile males [14] and induced to ovulate as previously described. *In vivo* fertilised embryos were collected from does slaughtered at 6 days of pregnancy by flushing uterine horns as previously described.

### RNA extraction, amplification and sample labelling

As the amount of RNA present in a single embryo is rather limited [15], for each experimental group (parthenotes and *in vivo* fertilised embryos) four independent pools consisting of seven embryos were produced. Total RNA was isolated using traditional phenol/chloroform extraction by sonication in the Trizol reagent (Invitrogen). Concentration, quality and integrity of RNA were evaluated by Bioanalyzer 2100 (Agilent Technologies). Afterwards, 150 ng of Total RNA were amplified and labelled using QuickAmp Labelling Kit (Agilent Technologies, Madrid, Spain), following the manufacturer's instructions, which employs a linear amplification method with T7 polimerase. Control embryo samples were labelled with Cyanine 5 dye (Cy5) and parthenote embryo samples with Cyanine 3 dye (Cy3). Excess dye was removed with the QIAquick PCR purification kit (QIAGEN, Madrid, Spain) and dye incorporation and concentration were determined using the microarray setting on the Nanodrop 1000.

### Hybridisation, washing and scanning of Microarrays

Equal amounts of Cy3 and Cy5 labelled samples (825 ng) were mixed with 10× Blocking Agent and Fragmentation Buffer, and then 55 µL of the mixture were hybridised into the commercial microarray specific for rabbit (Rabbit 44× oligonucleotide array; cat: G2519F -020908, Agilent Technologies, Madrid, Spain). This microarray was manufactured using the Agilent 60-mer SurePrint technology, which represented sequences of Refseq, Unigene and Ensembl databases (specifically 12083 identifiers of genes corresponding to the ENSEMBL database). After 17 hours at 65°C, hybridised slides were washed and scanned using the Agilent DNA Microarray Scanner G2565B (Agilent Technologies, Madrid, Spain). The resulting images were processed using the Feature Extraction v.10 Software (Agilent Technologies, Madrid, Spain) with default parameters. Only microarrays which passed control quality tests of Feature Extraction Software were used in posterior analysis.

### Microarray data analysis

Filtering of problematic probes identified as flag outliers and identification of differentially expressed genes between both experimental groups were performed using the software GeneSpring v.11.5 (Agilent Technologies, Madrid, Spain). A non-supervised analysis of global gene expression was performed using the principal components analysis (PCA). To identify differentially expressed genes, we used the T-test with Benjamini and Hochberg multiple test correction implemented in the GeneSpring (Agilent Technologies). Probe sets were considered differentially expressed between two conditions if they had a false discovery rate (FDR) of p-value<0.05. Gene Ontology analysis and functional annotation of differentially expressed genes were performed by Blast2GO software v.2.5.1 with default parameters [16]. All data sets related to this study were deposited in NCBI's Gene Expression Omnibus [17] and are accessible through GEO Series accession number GSE41043.

### Real-time qPCR

To validate the microarray results obtained, six genes (*IMPACT*; *SMARCA2*: SWI/SNF related matrix associated actin dependent regulator of chromatin subfamily A member 2; *EMP1*: Epithelial membrane protein 1; *DPY30*; *CALC*: calcitonin gene-related peptide variant 1; *SCGB1A1*: secretoglobin family 1A member 1) that showed a significant difference between experimental groups were selected and analysed in twelve independent pool samples (microarray samples plus additional pools). To prevent DNA contamination, one deoxyribonuclease treatment step (gDNA Wipeout Buffer, Qiagen Iberia S.L, Madrid, Spain) was performed from total RNA (1000 ng). Reverse transcription was then carried out using the Reverse Transcriptase Quantitect kit (Qiagen Iberia S.L, Madrid, Spain) according to the manufacturer's instructions. Real-time qPCR (RT-qPCR) reactions were conducted in an Applied Biosystems 7500 (Applied Biosystems, Foster City, CA). Every PCR was performed with 5 µL of 1/10 diluted cDNA of each sample used in each reaction in a final volume of 20 µL of 10 µL of SYBR Green Master Mix (Applied Biosystems) and 200 nM of forward and reverse primers (list of RT-qPCR primers is shown in Table 1). The PCR protocol included an initial step of 50°C (2 min), followed by 95°C (10 min) and 40 cycles of 95°C (15 sec) and 60°C (1 min). After RT-qPCR, a melting curve analysis was performed by slowly increasing the temperature from 65°C to 95°C, with continuous recording of changes in fluorescent emission intensity. Serial dilutions of cDNA pool made from several samples were run in triplicate to assess PCR efficiency and decide which dilution to use for unknown samples. Target and reference genes in unknown samples were run in duplicate. Non-template controls (cDNA was replaced by water) for each primer pair were run in all plates. A ΔΔC_t_ method adjusted for PCR efficiency was used [18], employing the geometric average of *H2AFZ* and *GAPDH* as normalisation factor [19] and relative expression of cDNA pooled from various samples was used as a calibrator. The products of RT-qPCR were confirmed by ethidium bromide-stained 2% agarose gel electrophoresis in 1× Bionic buffer.

**Table 1:** Information on primers used for real-time qPCR.

Gene	Accession number	Sequence 5′→3′	Fragment size (pb)	Efficiency (%)	Correlation (R^2^)
*IMPACT*	ENSOCUT00000013903	GCGTCTTCTTCACCTCATGG	116	104.8	0.99
		TGTTTCTTGGCACAGTTGTTGA			
*SMARCA2*	ENSOCUT00000006331	AATCCGCAACCACAAGTAAC	113	103.1	0.99
		GAACACTGACTGTAAGACGAT			
*EMP1*	ENSOCUT00000021095	AATGTTGGTGTTACTGGCTG	110	100.2	0.98
		GATGCGTTAATAGAGTCTGAA			
*SCGB1A1*	ENSOCUT00000014246	CCAGTTACGAGACATCCCTGA	155	93	0.99
		CATACACAGTGGGCTCTTCACT			
*DPY30*	ENSOCUT00000021095	GCAGAGAACCCTCATTCTGAG	148	98.4	0.99
		CGCACAACTGTCTGATCCTGGT			
*CALC*	ENSOCUT00000003074	GCTAGAGACTGAGGGCTCCA	124	90.8	0.99
		CACGAAGTTGCTCTTCACCA			
*H2AFZ*	AF030235	AGAGCCGGCTGCCAGTTCC	85	98.8	1
		CAGTCGCGCCCACACGTCC			
*GAPDH*	L23961	GTTCTTCTCGTGCAG	144	93.1	1
		ATGGATCATTGATGGCGACAACAT			

*H2AFZ*: H2A histone family member Z [35]; *GAPDH*: glyceraldehyde-3-phosphate dehydrogenase [36]; *SMARCA2*: SWI/SNF related, matrix associated, actin dependent regulator of chromatin, subfamily a, member 2; *EMP1*: Epithelial membrane protein 1; *CALC*: calcitonin gene-related peptide variant 1; *SCGB1A1*: secretoglobin family 1A member 1).

### Statistical Analysis

Data were analysed using the Statgraphics version Plus 5.1 (Statistical Graphics Co., Rockville, MD, USA,) software package. The relative expression data were analysed using General Linear Model (GLM). For *SMARCA2* a Neperian logarithmic transformation was done before analysis for data normalisation. Differences in mean values were tested using ANOVA followed by a multiple pair wise comparison using t-test. Differences of p<0.05 were considered to be significant.

## Results

### Parthenote embryo production and blastocyst recovery

From the total of 369 oocytes activated and transferred to recipient does, 49 blastocysts properly developed were recovered at day 6 post-activation (13.3%). Sixty-four *in vivo* fertilised blastocysts were recovered at day 6 post-insemination (88.9% related to ovulation rate, estimated as the number forming corpora lutea).

### Gene expression profiling and validation by real-time qPCR

**Figure 1:**
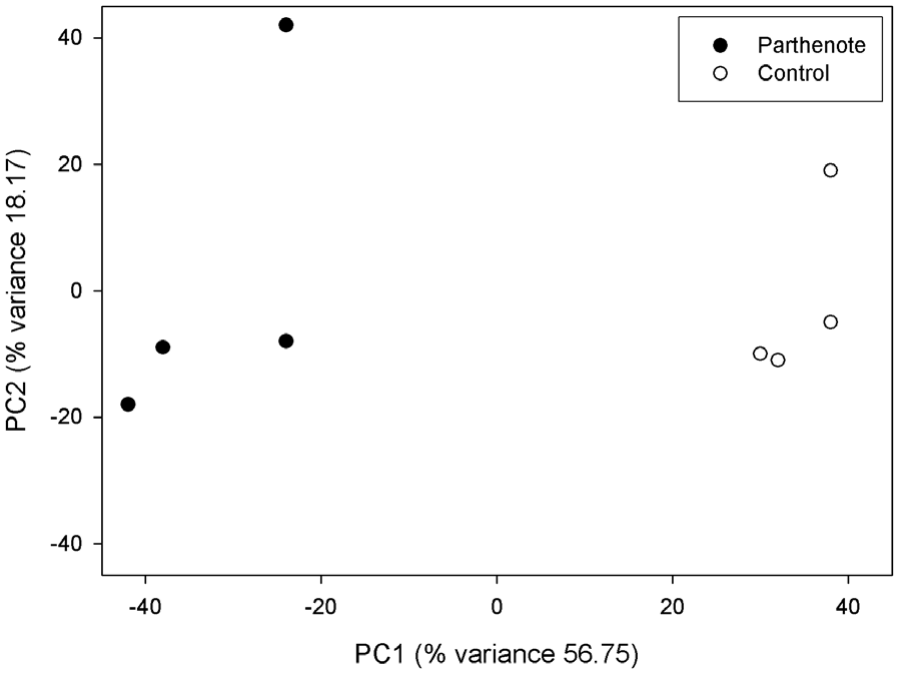
Principal Component Analysis (PCA) of microarray data. Principal Component Analysis (PCA) of microarray data. PCA two-dimensional scatter plot represent the differential gene expression patterns of frozen and control embryos. Axis: X = PC1: PCA Component 1 (56.75% variance); Y = PC2: PCA Component 2 (18.17% variance).

PCA showed that samples from the same group clustered together (Figure 1). Analysis of expression data identified a total of 2541 differentially expressed transcripts between 6-day-old parthenotes and *in vivo* fertilised embryos. Among these, 1185 were upregulated whereas the 1356 remaining transcripts were downregulated. Table 2 shows a classification of differentially expressed transcript probes based on fold-changes. Specifically, parthenogenetic blastocysts exhibited changes in the expression of 92 genes, of which 16 had lower expression and 76 showed higher expression than *in vivo* fertilised embryos using a minimal 3-fold change as a cut-off. The lists of the upregulated and downregulated genes in the parthenogenetic blastocysts are shown in Table 3 and 4, respectively.

**Table 2:** Classification of differentially expressed transcript probes based on fold changes.

	p-value		
Fold-change	<0.05	<0.02	<0.01
All	5790	881	20
>1.1	5547	870	20
>1.5	1606	363	14
>2.0	557	167	12
>3.0	199	67	8

**Table 3:** Genes upregulated by at least three-fold in parthenogenetic late blastocysts.

Gene symbol/probe	Gene accession	Gene name	Fold-change
**A_04_P030002**	C84254		−21,46
**MECOM**	ENSOCUT00000010173	MDS1 and EVI1 complex locus	−13,21
**Q95LB3**	ENSOCUT00000015946	Development promoting factor Oviductal glycoprotein 1	−14,60
**SAA1**	NM_001082327	Serum amyloid protein A	−13,32
**C20orf85**	ENSOCUT00000012758	Chromosome 20 open reading frame 85	−11,38
**MLF1**	ENSOCUT00000013546	Myeloid leukaemia factor 1	−10,39
**RTP4**	ENSOCUT00000007680	Receptor (chemosensory) transporter protein 4	−9,56
**SCGB1A1**	NM_001082237	Secretoglobin, family 1A, member 1 (uteroglobin)	−9,51
**CCDC153**	ENSOCUT00000016944	Coiled-coil domain containing 153	−9,28
**ADO**	ENSOCUT00000013986	Aldehyde oxidase	−8,57
**A_04_P017348**	DN886936		−8,19
**SULT1E1**	ENSOCUT00000005024	Sulfotransferase family 1E, oestrogen-preferring, member 1	−7,54
**C1orf189**	ENSOCUT00000016457	Chromosome 1 open reading frame 189	−7,02
**S100A4**	ENSOCUT00000008641	S100 calcium binding protein A4	−6,20
**A_04_P016580**	X00412		−7,46
**ZBTB20**	ENSOCUT00000004232	Zinc finger and BTB domain containing 20	−7,45
**SORBS2**	ENSOCUT00000005820	Sorbin and SH3 domain containing 2	−6,73
**SMARCA2**	ENSOCUT00000006331	SWI/SNF related, matrix associated, actin dependent regulator of Chromatin, subfamily a, member 2	−6,21
**B7NZD6**	ENSOCUT00000003373	Selenium binding protein 1	−6,45
**CCL2**	NM_001082294	Chemokine (C-C motif) ligand 2	−5,62
**EMP1**	NM_001082357	Epithelial membrane protein 1	−6,07
**CAPS**	NM_001082644	Calcyphosine	−5,54
**A_04_P033277**	EB380127		−5,79
**SPINK1**	ENSOCUT00000001659	Serine peptidase inhibitor, Kazal type 1	−5,20
**ANXA1**	ENSOCUT00000015491	Annexin A1	−5,13
**S100A14**	ENSOCUT00000002741	S100 calcium binding protein A14	−5,29
**CCL20**	ENSOCUT00000000868	Chemokine (C-C motif) ligand 20	−4,74
**PLAU**	NM_001082011	Plasminogen activator, urokinase	−5,03
**C11orf70**	ENSOCUT00000006101	Chromosome 11 open reading frame 70	−4,91
**FANK1**	ENSOCUT00000008774	Fibronectin type III and ankyrin repeat domains 1	−4,65
A_04_P092437	ENSOCUT00000013589		−4,81
**MYL4**	ENSOCUT00000010827	Myosin, light chain 4, alkali; atrial, embryonic	−4,81
**A_04_P035022**	ENSOCUT00000005248		−4,73
**IL1R1**	NM_001082770	Interleukin 1 receptor, type I	−4,54
**SLC16A7**	ENSOCUT00000003051	Solute carrier family 16, member 7 (monocarboxylic acid transporter 2)	−4,18
**A_04_P044537**	ENSOCUT00000012839		−4,35
**CLUS**	ENSOCUT00000005984	ClusterinClusterin beta chain Clusterin alpha chain	−4,34
**NPY**	ENSOCUT00000010758	Neuropeptide Y	−4,31
**A_04_P016911**	DN884335		−4,24
**CAV1**	NM_001111072	Caveolin 1, caveolae protein	−4,12
**TNNI1**	ENSOCUT00000010422	Troponin I, slow skeletal muscle	−4,01
**ARAP2**	ENSOCUT00000015685	ArfGAP with RhoGAP domain, ankyrin repeat and PH domain 2	−3,97
**DYNLRB2**	ENSOCUT00000008571	Dynein, light chain, roadblock-type 2	−3,92
**ALAS2**	ENSOCUT00000013600	Aminolevulinate, delta-, synthase 2	−3,85
**HECW1**	ENSOCUT00000008000	HECT, C2 and WW domain containing E3 ubiquitin protein ligase 1	−3,76
**SCMC1**	ENSOCUT00000012809	Calcium-binding mitochondrial carrier protein SCaMC-1	−3,75
**OCA2**	ENSOCUT00000003517	Oculocutaneous albinism II	−3,56
**CTBS**	ENSOCUT00000003057	Chitobiase, di-N-acetyl-	−3,71
**A_04_P016912**	DN884335		−3,71
**A_04_P060497**	ENSOCUT00000006983		−3,69
**GPIIIa**	NM_001082066	Glycoprotein IIIa	−3,47
**B3GS73**	ENSOCUT00000007932	CCL28	−3,49
**CD48**	ENSOCUT00000013544	CD48 molecule	−3,60
**LIPC**	ENSOCUT00000001646	Hepatic triacylglycerol lipase	−3,45
**GST**	ENSOCUT00000011951	Glutathione S-transferase	−3,44
**SLC25A23**	NM_001082777	Solute carrier family 25 (mitochondrial carrier ; phosphate carrier), Member 23 nuclear gene encoding mitochondrial protein	−3,44
**LRRIQ1**	ENSOCUT00000017528	Leucine-rich repeats and IQ motif containing 1	−3,43
**ST3GAL5**	ENSOCUT00000010127	ST3 beta-galactoside alpha-2,3-sialyltransferase 5	−3,42
**LMO2**	ENSOCUT00000001532	LIM domain only 2 (rhombotin-like 1)	−3,37
**O97770**	ENSOCUT00000016899	Titin	−3,37
**A_04_P013028**	K02441		−3,22
**CTBS**	ENSOCUT00000003057	Chitobiase, di-N-acetyl-	−3,32
**MYL3**	ENSOCUT00000012390	Myosin, light chain 3, alkali; ventricular, skeletal, slow	−3,32
**PPIL6**	ENSOCUT00000006037	Peptidylprolyl isomerase (cyclophilin)-like 6	−3,31
**A_04_P033242**	EH792761		−3,26
**SCMC1**	ENSOCUT00000012809	Calcium-binding mitochondrial carrier protein SCaMC-1	−3,16
**A_04_P054532**	ENSOCUT00000000433		−3,20
**GPRC5A**	ENSOCUT00000016550	G protein-coupled receptor, family C, group 5, member A	−3,16
**TTC18**	ENSOCUT00000007154	Tetratricopeptide repeat domain 18	−3,16
**MAMDC2**	ENSOCUT00000000271	MAM domain containing 2	−3,16
**C1RL**	ENSOCUT00000014491	Complement component 1, r subcomponent-like	−3,15
**RSPH9**	ENSOCUT00000005536	Radial spoke head 9 homolog (Chlamydomonas)	−3,11
**A_04_P004519**	ENSOCUT00000011542		−3,06
**A_04_P034797**	ENSOCUT00000008808		−3,04

Genes are tabulated in the descending order of the fold-change values. Transcripts without annotation were identified by probe set ID.

**Table 4:** Genes downregulated by at least three-fold in parthenogenetic late blastocysts.

Gene/probe	Gene accession	Gene name	Fold-change
**A_04_P013564**	EB375829		51,83
**SNRPN**	NM_001082714	Small nuclear ribonucleoprotein polypeptide N	48,40
**CALC**	ENSOCUT00000003074	Calcitonin gene-related peptide variant 1	7,54
**TAC1**	NM_001101698	Tachykinin, precursor 1	7,26
**MS4A13**	ENSOCUT00000015913	Membrane-spanning 4-domains, subfamily A, member 13	6,67
**A_04_P017715**	EB373964		6,55
**IMPACT**	ENSOCUT00000013903	Protein IMPACT	4,58
**KRTCAP3**	ENSOCUT00000004321	Keratinocyte associated protein 3	4,35
**A_04_P085877**	ENSOCUT00000003190		3,58
**KPB2**	ENSOCUT00000013796	Phosphorylase b kinase regulatory subunit alpha, liver isoform	3,47
**A_04_P035497**	ENSOCUT00000016846		3,25
**DPY30**	ENSOCUT00000017876	Dpy-30 homolog	3,23
**RIT1**	ENSOCUT00000006374	Ras-like without CAAX 1	3,16
**Q8SQB7**	ENSOCUT00000001908	Inducible nitric oxide synthase	3,13
**CXCR7**	ENSOCUT00000010904	Chemokine (C-X-C motif) receptor 7	3,10
**PON3**	ENSOCUT00000002011	Serum paraoxonase/lactonase 3	3,07

Genes are tabulated in the descending order of the fold-change values. Transcripts without annotation were identified by probe set ID.

All genes selected to validate the microarray analysis exhibited expression patterns in line with previous results. Similarly, the three genes that exhibited lower expression in parthenotes in the microarray experiment (*MPACT*, *DPY30* and *CALC*) also showed decreased expression by RT-qPCR (Table 5), while three genes showing higher expression in parthenogenetic blastocysts by the microarray analysis (*SCGB1A1*, *EMP1* and *SMARCA2*) also exhibited increased expression by RT-qPCR (Table 5). Comparisons between fold-change of results for RT-qPCR and microarray are shown in Table 5. The PCR experiments reproduced the microarray profiling for selected genes, although fold changes differed between RT-qPCR and microarray, which can be explained by different probes used for RT-qPCR and microarray [20].

**Table 5:** Real-time quantitative PCR assay for six randomly selected genes.

	Relative expression (a.u.)		Fold change	
Gene	Fertilised embryos	Parthenote embryos	RT-qPCR	Microarray
*IMPACT*	0.82±0.16^a^	0.004±0.21^b^	7.68	4.58
*DPY30*	1.24±0.14^a^	0.27±0.18^b^	2.20	3.23
*CALC*	0.56±0.04^a^	0.14±0.05^b^	2.00	7.54
*SCGB1A1*	0.04±0.22^a^	1.25±0.25^b^	−4.96	−9.51
*EMP1*	0.48±1.99^a^	8.37±1.99^b^	−4.12	−6.07
*SMARCA2*	0.16±0.51^a^	1.76±0.51^b^	−3.45	−6.21

*SMARCA2*: SWI/SNF related, matrix associated, actin dependent regulator of chromatin, subfamily a, member 2; *EMP1*: Epithelial membrane protein 1; *CALC*: calcitonin gene-related peptide variant 1; *SCGB1A1*: secretoglobin family 1A member 1). Relative expression values are shown in arbitrary units (a.u), expressed by the mean value ± standard error means. Letters with different superscripts are significantly different (P<0.05). RT-qPCR fold changes were obtained by calculation of log2 transformed ratio of relative expression for each gene. Microarray fold changes were obtained by log2 transformed probe intensities for each gene.

**Figure 2:**
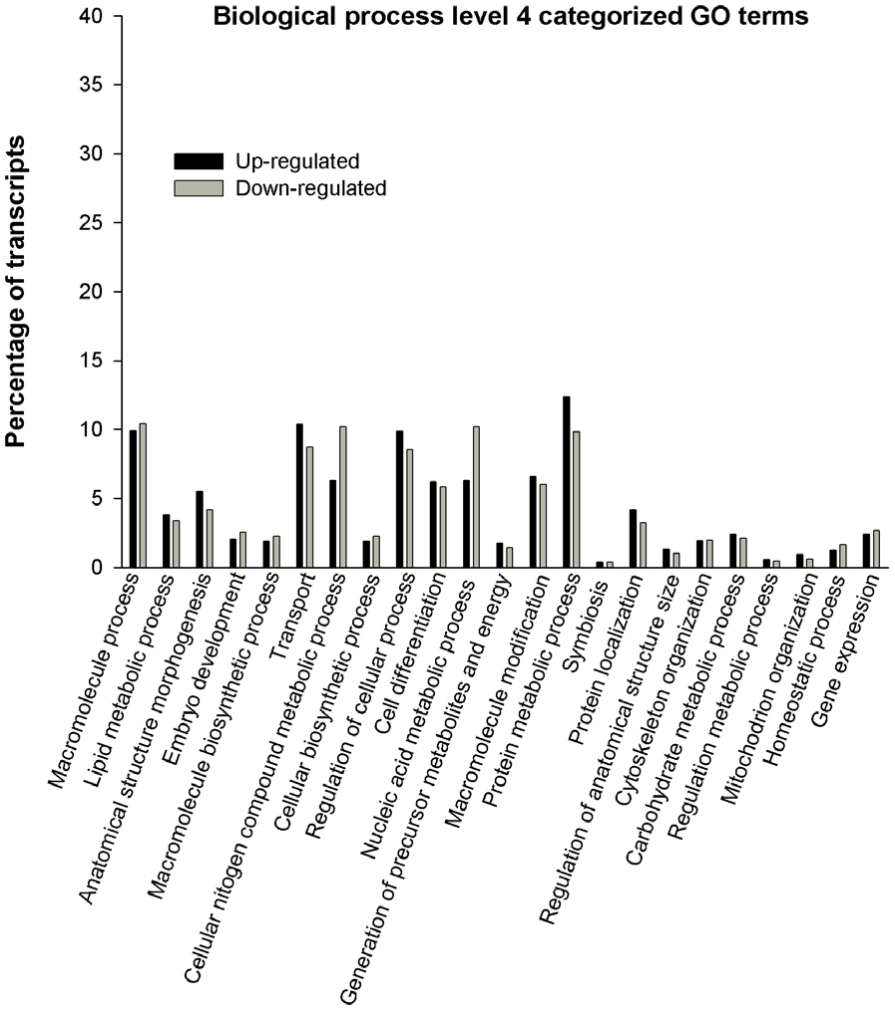
Gene Ontology (GO) bar chart of differentially expressed genes between parthenotes and fertilised embryos. Gene Ontology (GO) bar chart of differentially expressed genes between parthenotes and in vivo fertilised embryos. Genes upregulated and downregulated in parthenotes embryos that are categorised by GO term “Biological process” level 4.

**Figure 3:**
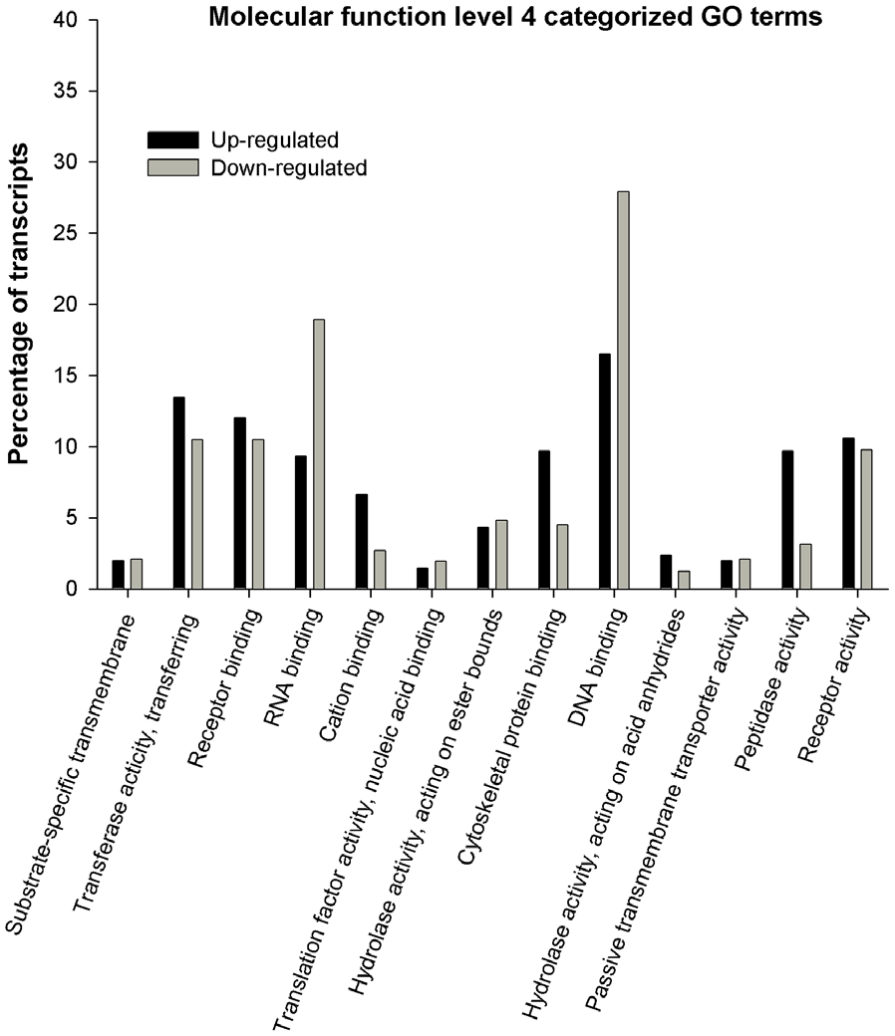
Gene Ontology (GO) bar chart of differentially expressed genes between parthenotes and fertilised embryos. Gene Ontology (GO) bar chart of differentially expressed genes between parthenotes and in vivo fertilised embryos. Genes upregulated and downregulated in parthenotes embryos that are categorised by GO term “Molecular function” level 4.

**Figure 4:**
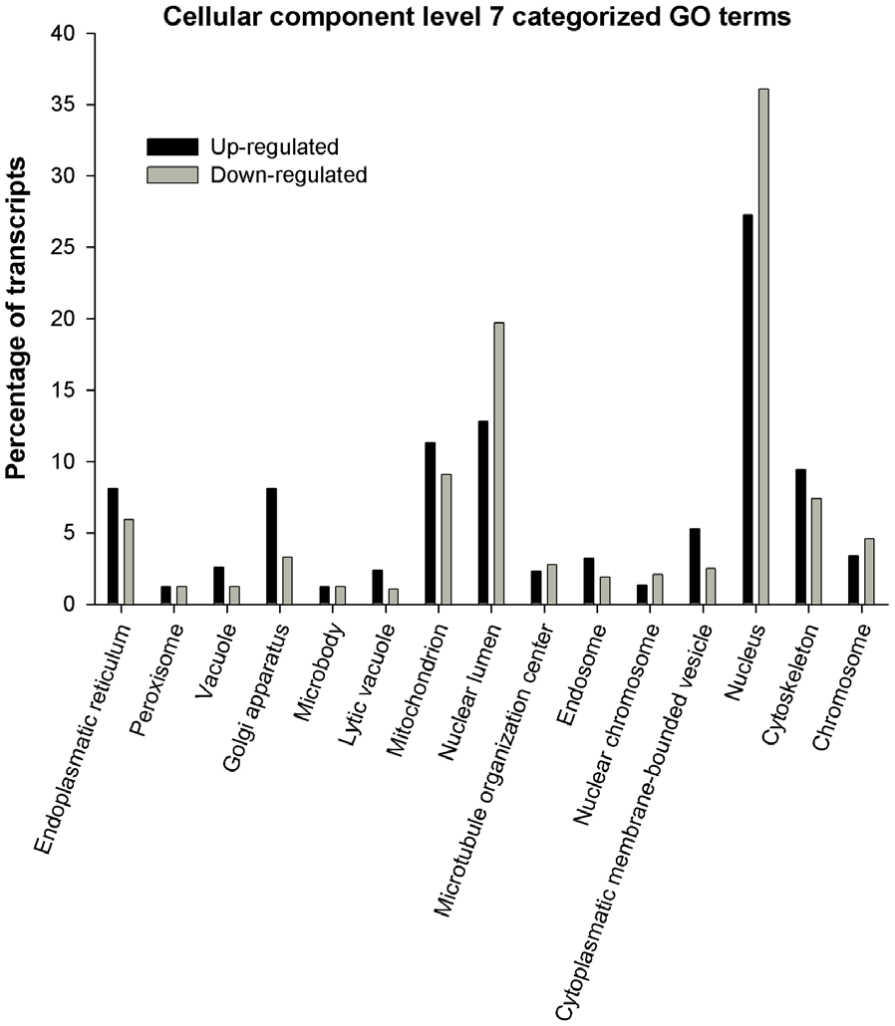
Gene Ontology (GO) bar chart of differentially expressed genes between parthenotes and fertilised embryos. Gene Ontology (GO) bar chart of differentially expressed genes between parthenotes and in vivo fertilised embryos. Genes upregulated and downregulated in parthenotes embryos that are categorised by GO term “Cellular Component” level 7.

Biological process, molecular function and cellular component vocabulary items assigned to upregulated and downregulated genes in parthenote embryos are shown in Figures 2, 3, and 4 respectively. For Biological Process, the most represented categories of altered genes were those related to cellular macromolecule process, transport, regulation of cellular process, protein metabolic process, nucleic acid metabolic process and macromolecule modifications (Figure 2). As far as molecular function is concerned, the most represented GO terms were DNA and RNA binding, receptor binding and transferase activity (Figure 3). Finally, main annotations for cellular components are those related to mitochondrion, nuclear lumen, nucleus and cytoskeleton (Figure 4).

### Putatively imprinted genes

In parthenote embryos expression of paternally expressed imprinted genes is not expected, since both alleles are of maternal origin. We extracted information probes from the microarray data that detected known or putative imprinted genes (Catalogue of Imprinted Genes; http://igc.otago.ac.nz/home.html). Six of the genes which appear as most specifically upregulated or downregulated in the microarray have previously been annotated as imprinted genes. *GRB10* and *ATP10A* were upregulated in parthenotes, as expected because the maternal allele is the one expressed, while *ZNF215*, *NDN*, *IMPACT* and *SFMBT2* were downregulated according to the paternal allele expression. Furthermore, 26 other genes of the microarray which were significantly different in parthenote embryos, also shown to have at least one member of that gene family imprinted in other species (Table 6).

**Table 6:** Putative imprinted genes differentially expressed in parthenogenetic late blastocysts identified as family members at Catalogue of Imprinted Genes (http://igc.otago.ac.nz/home.html).

	Family members genes name	
Imprinted gene	Upregulated	Downregulated
*SLC22A2, SLC22A3, SLC22A8, SLC22A18S*		*SLC22A5, SLC22A17*
*AWT1,WT1-AS*		*SWT1*
*IGF2*	*IGF2BP2*	*IGF2BP3*
*RB1*	*RB11A*	
*L3MBTL*	*L3MBTL2*	*L3MBTL1*
*PPP1RGA*		*PPP1CC*
*ASB4*	*ASB8*	*ASB3*
*KLF14*	*KLF16, KLF12*	*KLF3, KLF4*
*NAP1L5*	*NAP1L1*	
*UPS29*	*USP2, USP4, USP25, USP53*	*USP7, USP15, USP22, USP28, USP34USP40, USP43, USP46, USP48*
*ZFP264, ZFP127*		*ZFP36, ZFP57, ZFP62, ZFP90*
*PEC2, PEC3*		*PECR*
*NCCR*		*NCCRP1*
*UBE3A*	*UBE3B, UBE4B*	
*TSPAN32*	*TSPAN5, TSPAN12, TSPAN13*	*TSPAN1N, TSPAN14, TSPAN31*
*TNFRSF23*		*TNFRSF1A*
*ANO1*		*ANO6*
*INPP5F-V2*	*INPP1, INPP4B*	
*RASGRF1*	*RASGEF1B, RASGRP3*	*RASGRP1, RASGRP2*
*COMMD1*	*COMMD3, COMMD5*	*COMMD2, COMMD7, COMMD8*
*HTR2A*		*HTRA4*
*FBXO40*	*FBXO15, FBXO32, FBXO48*	*FBXO4, FBXO5, FBXO25, FBXO38, FBXO42*
*SNRPN*		*SNRPPA1, SNRPB2*
*PRIM2*		*PRIM1*
*CDKN1C*	*CDKN1A, CDKN1B, CDKN3*	
*SASH2*		*SASH1*

## Discussion

Our results demonstrated that parthenotes and *in vivo* fertilised rabbit blastocysts cultured under *in vivo* conditions differ notably in gene expression. Up till now, few works have analysed transcriptome differences between parthenotes and fertilised embryos [20], [21], [22]. However, these works were carried out with parthenote embryos developed *in vitro* and *in vitro* cultured fertilised embryos. It is well documented that embryos developed under *in vitro* environment are still not comparable with *in vivo* embryos [23], as post-fertilisation culture environment is a determinant for adequate embryonic development [4], [24]. For example, one of the most critical time points of preimplantation embryogenesis is the major embryonic genome activation at which the embryo switches from using the mRNA and proteins derived from the maternal genome to those resulting from de novo transcription from the embryonic genome [25]. During that time, availability of transcription factors, which are regulated by cell cycle-dependent mechanisms, is required [26]. These mechanisms are strongly influenced by a change in environmental conditions and subsequently affect the embryonic development, with potentially severe effects on foetal, prenatal and postnatal viability [27]. Corcoran et al. [20] found that a total of 384 genes were differentially expressed between *in vivo* and *in vitro* derived blastocysts, the vast majority of them (almost 85%) being downregulated in *in vitro* developed embryos. Likewise, the effects of developmental environment on mRNA expression in parthenogenetic embryos have also been described [11] this way. To our best knowledge, this is the first report that compared the genome-wide gene expression profiles between rabbit parthenogenetic blastocysts and fertilised blastocysts developed *in vivo*.

Microarray analysis of parthenotes and fertilised embryos developed *in vitro* indicated differences in expression of 749 genes from mouse with 1.8 fold-changes as a cut-off [20], 24 genes for early embryos and 5 for expanded embryos from bovine with 1.5 fold-changes as a cut-off [22] and 56 genes from buffalo with 1.4 fold-changes as a cut-off [21]. In this study, we observed that 1606, 557 and 199 microarray probe signals were changed in the parthenogenetic blastocyst using a minimum of 1.5, 2.0 and 3.0 fold-changes as a cut-off, respectively. The 199 probe signals represent 92 genes, of which 16 had lower expression and 76 showed higher expression in parthenotes than fertilised embryos, developed *in vivo*. In the present study, in terms of biological process categories, slight differences are observed between transcript percentage of up and downregulated genes. However, the main categories altered, related to transport and protein metabolic process, comprise more upregulated than downregulated genes. Genes with high fold-changes such as *BZND6*, *ANXAL*, *MYL4* are involved in transport, while protein metabolic process includes genes such as *ClUS*, *PPIL6* or *CIRL*. In contrast, regarding molecular function and cellular components, a higher percentage of downregulated transcripts are comprised. In this case, the main altered categories are those related to DNA and RNA binding, both located in cellular nucleus and involving genes such as *GTF2B* (general transcription initiation factor IIb; X), *CHURC1* (Churchill domain containing 1), *XRCC2* (DNA repair protein XRCC2), *HNRNPD* (heterogeneous nuclear ribonucleoprotein D), *SAFB2* (scaffold attachment factor B2) or *NEIL3* (nei endonuclease VIII-like 3) among others. So, these results suggest a great deficiency of the machinery associated with transcription and translation which might hinder basic cell functioning and thereby pre-implantatory development of parthenogenotes. Similar results of the main categories altered in biological processes have been observed before in gene expression profile studies of *in vitro* developed parthenotes. Processes such as proteolysis, peptidolysis, protein amino acid phosphorylation and cell transport showed to be the most representative upregulated in parthenotes, while nucleic acid binding and metabolic process were representative of the higher percentage of donwregulated transcripts in parthenotes [20], [21].

To date, more than 100 imprinted genes have been identified in mice and many of them are also imprinted in humans [29]. In livestock animals, imprinted genes have also been identified [30], [31], [32], [33]. However, to our best knowledge, few genes have been identified as subject to genomic imprinting in rabbit. All imprinted genes show either maternal-specific or paternal-specific mono-allelic expression, and their proper expression is essential for normal development, foetal growth, nutrient metabolism and adult behaviour [34]. We extracted informative probes from the microarray data that detected known or putative imprinted genes (Catalogue of Imprinted Genes; http://igc.otago.ac.nz/home.html). Of the 32 putative genes analysed in this manner (table 6), 6 were identified as conserved between rabbits, humans and mice; they included *GRB10*, *ATP10A*, *ZNF215*, *NDN*, *IMPACT* and *SFMBT2*. GRB10, SNRPN and CDKN1 were also shown to be imprinted in a previous work carried out with *in vitro* developed parthenotes in mouse [20]. In fact, the use of microarrays to analyse imprinted genes provided results in the same direction as quantitative allelic pyrosequencing (QUASEP) analysis [30].

In conclusion, the resulting findings of this study revealed that even under the best developmental conditions, parthenogenetic and fertilised embryos at the late blastocyst stage are different, with at least 92 genes significantly and differentially expressed. These differences have been shown to affect basic functions such as DNA and RNA binding, nucleus, mitochondrion and transport, among others. ESCs may inherit the blastocyst level of transcripts, and the alterations observed in parthenogenetic embryos could therefore be maintained in pESCs derived from them. These alterations in gene expression call for further studies to evaluate whether and to what extent these modifications are unfavourable for ESC establishment and successive transplantation therapies. Furthermore, this work represents the first approach to the study of imprinted genes in rabbit. Hence, future research into imprinted genes might also include rabbits as alternative model systems.
